# Risk of dengue virus infection according to serostatus in individuals from dengue endemic areas of Mexico

**DOI:** 10.1038/s41598-020-75891-z

**Published:** 2020-11-04

**Authors:** I. Y. Amaya-Larios, R. A. Martínez-Vega, F. A. Diaz-Quijano, E. Sarti, E. Puentes-Rosas, L. Chihu, J. Ramos-Castañeda

**Affiliations:** 1 Centro de Investigaciones Sobre Enfermedades Infecciosas, Instituto Nacional de Salud Publica, Av Universidad 655, Santa Maria Ahuacatitlan, 62100 Cuernavaca, Morelos Mexico; 2 Consejo Nacional de Ciencia y Tecnología, Mexico, Mexico; 3 Universidad de Santander, Bucaramanga, Colombia; 4 Organización Latinoamericana para el Fomento de la Investigación en Salud, Bucaramanga, Colombia; 5 Faculdade de Saúde Pública, Universidade de São Paulo, São Paulo, Brazil; 6 Sanofi Pasteur México, CDMX, Mexico; 7 Center for Tropical Diseases, University of Texas-Medical Branch, Galveston, USA

**Keywords:** Infectious diseases, Viral infection

## Abstract

The variability in the host immune response directed against dengue virus (DENV) has demonstrated the need to understand the immune response associated with protection in incident infection. The objective was to estimate the association between serostatus and the risk of incident DENV infection. We used a prospective study from 2014 to 2016 in the localities of Axochiapan and Tepalcingo, Morelos, Mexico. We recruited 966 participants, of which, according to their infection history registered were categorized in four groups. To accomplish the objectives of this study, we selected to 400 participants older than 5 years of age were followed for 2.5 years. Blood samples were taken every 6 months to measure serological status and infection by ELISA. In individuals with at least two previous infections the risk of new infection was lower compared to a seronegative group (hazard ratio adjusted 0.49, 95% CI 0.24–0.98), adjusted for age and locality. Therefore, individuals who have been exposed two times or more to a DENV infection have a lower risk of re-infection, thus showing the role of cross-immunity and its association with protection.

## Introduction

Dengue is a viral disease transmitted by *Aedes* mosquitoes and is considered a public health problem worldwide^1^. The clinical spectrum of infection by any of the four serotypes of dengue virus (DENV) is wide because it may present as an asymptomatic infection, a symptomatic infection or sometimes a lethal infection^2^.

In 2014, 40% of the world’s population was at risk of being infected, with the number estimated as between 50 and 100 million infections per year by any of the four serotypes (DENV-1 to DENV-4)^3^. However, Bhatt et al. estimated that the number of infections in the world is three times higher than that reported by the World Health Organization (WHO), with 294 million unapparent infections (95% CI 217–392) and 96 million apparent infections (95% CI 217–392)^4^.

The maintenance of disease transmission in tropical and subtropical countries depends on demographic factors as well as altitude, temperature, and humidity. Additionally, the immunity generated in the population against several DENV serotypes and the evolution of the virus itself are considered factors influencing transmission, contributing to periodicity in the onset of dengue cases^5^.

Humoral immunity may increase the risk of showing the severe form of dengue, as suggested by several studies performed in Thailand, Indonesia, and Cuba that estimated titers of neutralizing antibodies related to protection and evaluated the association between the presence of preexisting neutralizing antibodies and severe cases^3,6–9^ .

On the other hand, several findings have been observed that conflict with the enhancement theory of the immune response. For example, in Peru, febrile cases reported in the postsecondary infection group compared to the primary and secondary infection groups dropped significantly by 93% for DENV-3, and 64% for DENV-4, showing that preexisting cross-reactive antibodies may decrease the risk of infection^10,11^.

Likewise, a study that evaluated the relationship between humoral immunity and protection found that the preexistence of titers of neutralizing homotypic antibodies is associated with protection against infections by DENV-1, DENV-2, and DENV-4^11^.

Endemic transmission of DENV is intense in Mexico, the force of infection has been estimated in approximately 0.02^12^. In the studied area, Axochiapan and Tepalcingo, Morelos, 79% of average seroprevalence occurs and the majority of the population have neutralizing antibodies against all serotypes after 10 years old^13^; therefore, In this area, all DENV serotypes have been circulated since first recorded in 1984, but DENV-1 has been predominate since 2009 with a limited circulation of DENV-2 since 2014^13^ (Supplementary Table S1) it is essential and necessary to evaluate the role of preexisting antibodies to define their protective or enhancing role in subsequent infections. Thus, in this study, we assessed the seroconversion dynamics in two Mexican endemic populations through the annual estimation of seroprevalence, and further estimated the association between previous serostatus and the risk of incident DENV infection between August 2014 and December 2016.

## Results

### Recruitment and characteristics of participants

**Figure 1:**
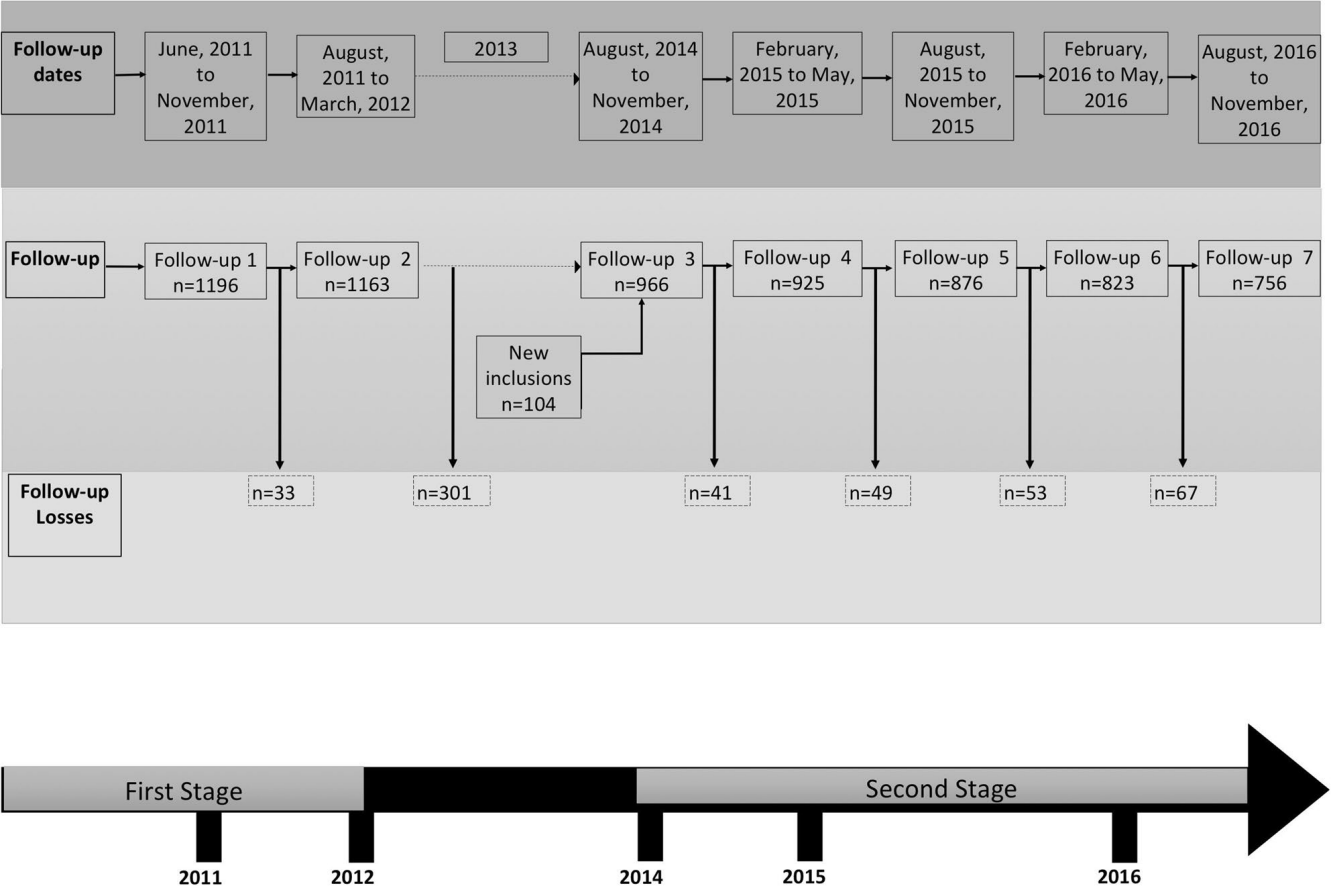
Follow-up of the cohort (2011–2016) and losses to follow-up.

The endemic population were studied in two stages, the first stage comprising 2010 and 2011 and it was reported elsewhere^13,14^.The recruitment for the second stage started in August 2014 and ended on November 2016, with a total of 7 follow-ups from 2011 to 2016. From the original cohort, 72% (n = 862/1196) were recruited with 104 new inclusions (n = 966). The total dropout registered from follow-up 3 to follow-up 7 was 21.7% (n = 210/966) (Fig. 1). The mean follow-up time for each stage from 2014 was approximately 6 months.

The average age of cohort participants included from the follow-up on August 2014 (follow-up 3) was 34.2 years, with ages ranging between 6 and 95 years. Approximately 70% of participants stated that they lived in the locality of Axochiapan, and most of them had access to healthcare (Table 1).

**Table 1:** Sociodemographic characteristics of the second stage of the cohort (2014).

Group	Total n = 966	Seronegative	Seropositive with at least two previous infections	Seropositive with at least one previous infections	Seropositive group without recent infection from 2011 to 2012					
		1 n = 90	2 n = 185	3 n = 125	4 n = 566					
**Characteristics**										
Age, years										
Mean ± SD	34.2 ± 16.45	19.5 ± 11.8	34.8 ± 21.9	22.2 ± 14.5	39.82 ± 18.0					
Median [range]	32 [6–95]	16 [8.5–84]	27.6 [8.4–88.6]	17 [6–80.2]	38.9 [6.3–95.8]					

	n	%	n	%	n	%	n	%	n	%
**Locality**										
Axochiapan	878	67.9	52	57.8	140	75.7	83	66.4	376	66.4
**Gender**										
Woman	579	60.0	39	43.3	74	40.0	63	50.4	211	37.3
**Access to healthcare**										
Health insurance	858	88.8	89	98.9	164	88.6	112	89.6	501	88.5
Not health insurance	106	11.0	1	1.1	21	11.4	13	10.4	64	11.3
Not evaluated	2	0.2	0	0.0	0	0.0	0	0.0	1	0.2

*SD* standard deviation.

### Seroprevalence

**Figure 2:**
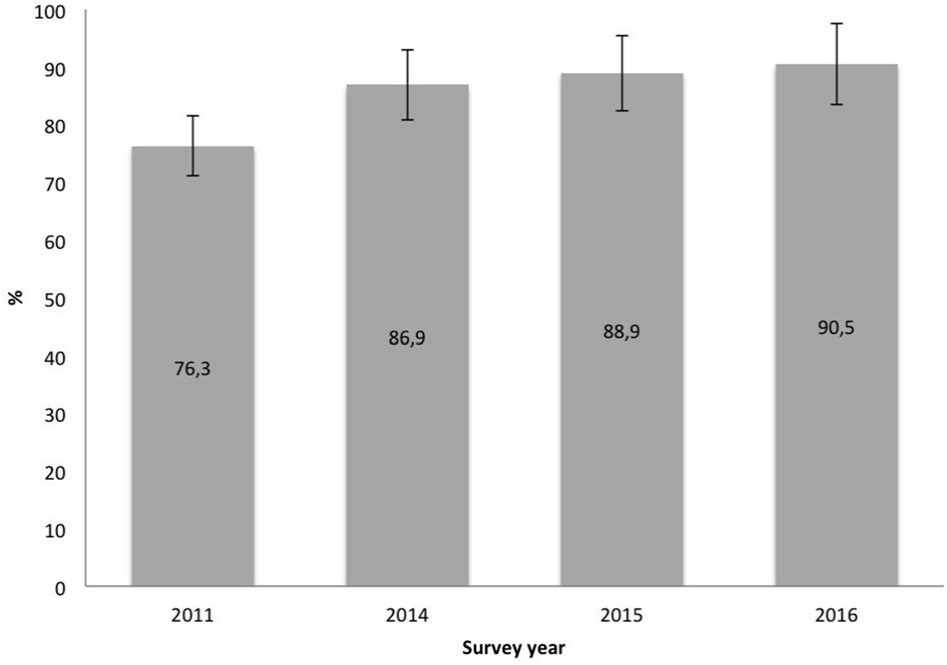
Seroprevalence of Axochiapan and Tepalcingo (2011–2016).

In 2016 the seroprevalence for DENV in the cohort was 90.5% (95% CI 92.2–95), which increased 14.2 percentage points from 2011 (Fig. 2).

### Incidence of recent infection by DENV

In the second stage of the cohort, 966 participants were included, of which, according to their infection history registered between 2011 and 2012 (first stage of the cohort), 125 were categorized in the seronegative group (Group 1), 185 participants in the seropositive with at least two previous infections from the follow-up in 2012 (Group 2), 90 participants in the seropositive with at least one previous infection (Group 3), and 566 participants in the seropositive group without recent infection (Group 4).

To fulfill the objective of the study, the infections of the participants belonging to groups 1–3 were diagnosed, because their history of infection was already known (n = 400). In these groups, during follow-ups 3–7, a total of 93 new infections were identified, including 7 reinfections. During the third follow-up, 44 infections were detected, including 11 in group 3 (average age 17.21 years, standard deviation [SD] 6.19), and the remaining infections belonged to the group 2 (average age of 32.3 years, SD 20.47 years).

During follow-ups 4–7, 48 infections were identified, with a total infection rate of 7.5 per 1000 person-years (95% CI of 5.66–9.96) (Table 2, Supplementary Table S2). 61.2% (n = 30) of the infections during follow-up 4–7 were asymptomatic. The rest, 18 infections, were considered symptomatic dengue infections because they stated that they had presented fever, by the telephone follow-up carried out between visits to their homes and they had serology compatible with recent infection; no virological diagnosis (NS1 detection or RT-PCR) of these patients was carried out and its clinical course was ambulatory. Additionally, one participant showed seroconversion based on dengue IgG-Indirect but was negative for IgG-Capture and IgM-Capture, and therefore was not considered as a recent infection, because the recent infection status could not be confirmed.

**Table 2:** DENV infection rate per 1000 person-years during follow-ups 4–7 (2014–2016).

Group	Type of infection	Person- years	Infections (n)	Incidence rate	95% confidence interval	
Seronegative	Primary infections	202.23	19	9.44	6.02	14.80
Seropositive with at least one previous infections	Secondary infections	155.00	15	9.62	5.80	15.95
Seropositive with at least two previous infections	Postsecondary infections	282.29	14	4.96	2.94	8.37
Total		639.52	48	7.50	5.66	9.96

In addition, 18 participants were identified as having maintained detectable IgM levels during 2 years of follow-up; therefore, they were not considered recent DENV infections. These participants will be evaluated in future studies to determine the causes underlying the continuing IgM level.

### Relationship between previous serostatus and incident DENV infection

Among participants with two or more previous infections (Group 2) in which additional infections were diagnosed, the infection risk was decreased by 51.4% on average compared to seronegative individuals, adjusted for age and locality. In contrast, we found no differential risk of infection during the follow-up for individuals with one prior infection compared to seronegative participants.

Additionally, it was observed that living in Axochiapan reduced the infection risk compared to inhabitants of Tepalcingo (p < 0.05) (Table 3). Proportionality assumptions of the Cox model were met for each covariate and for the overall model.

**Table 3:** Factors related to incident DENV infection in cohort participants from Axochiapan and Tepalcingo, Morelos (2014–2016).

	HR	Robust standard error	p >|z|	95% confidence interval	
**Group**					
Seronegative	1				
Seropositive with at least one previous infections	1.00	0.370	0.999	0.48	2.07
Seropositive with at least two previous infections	0.49	0.174	< 0.05	0.24	0.98
Age	1.01	0.007	0.156	1.00	1.02
**Locality**					
Tepalcingo	1				
Axochiapan	0.44	0.134	< 0.05	0.24	0.81

*HR* hazard ratio.

## Discussion

This work is notable for its long follow-up period, which allowed assessment of the seroconversion dynamics in these Mexican endemic populations. This assessment was achieved by estimating annual seroprevalence, as well as by calculating the protective effect of the previous seropositive state in decreasing recent infections by DENV in two Mexican endemic populations.

Our research studied two endemic localities in the South-Central region of the country to evaluate the role of humoral immunity against DENV. The results showed an increase of 14.7 percentage points in the seroprevalence between 2012 and 2016. This observed increase is attributable to DENV infections for two reasons: (1) only one case of zika was register by the local/country epidemiolocal surveillance system in the study area, this occurred at the beginning of the last follow-up; the case was not confirm by laboratory test (qRT-PCR) and no other case was reported in persons who lives around the case nor in the entire locality on 2016^15^. (2) The seroprevalence is the presence of IgG antibodies against DENV, and it is detected using a different diagnostic kit (indirect IgG) to determine recent infection (capture IgM/IgG ELISA); this distinction is relevant because by definition the antibodies detected in the indirect ELISA kit are cross-reactive and only report exposure to DENV in regions where only this flavivirus circulates; in regions where more than one flavivirus circulates, the diagnostic kit reports exposure to flaviviruses^16^. Thus, if there were active transmission of another flavivirus in addition to DENV, the seroprevalence should be significantly increased; however, as can be seen (Fig. 2) there is no significant change in the prevalence of antibodies against DENV (flavivirus) in the years before to 2016, compared to that year in which a zika case was reported. On the other hand, this increment reflects the endemic nature of the population, in that transmission occurs despite the stable implementation of multiple vector control measures^17–19^ .

Likewise, we observed that seropositivity rates in endemic regions generally increased with age, consistent with what has been reported in other countries in Latin America and Asia. Age is a surrogate of time of exposure, meaning that older people have a higher probability of having been infected more than once with DENV, leading to an increase in the titers of neutralizing antibodies against the virus that may act as a boost in immunity^20–25^.

In our study, as well as the study of a pediatric cohort in Nicaragua, the results suggested that the duration of cross-immunity is approximately 2 years and is associated with non-severe dengue infection; after that time, the probability of a symptomatic infection increases^7,26^. Additionally, the cumulative cross-immunity response over the years of exposure confers protection to the individuals and may be one explanation for the lower frequency of post-secondary infections in endemic populations; therefore, it has been proposed that the serostatus prior to infection may determine the clinical form of disease, and in fact pre-existing cross-reactive antibodies may be mainly a protective factor, corroborating the importance of heterotypic humoral immunity and the duration of cross-immunity in response to a natural infection by DENV^7,10,26–29^ .

Regarding the geographical distribution of the study localities, our results showed a reduced infection risk for individuals living in Axochiapan compared to those living in Tepalcingo. This result is different from that reported in the first stage of the cohort, in which the infection risk was higher for inhabitants of Axochiapan (RR 1.64, 95% CI 1.22–2.21)^23^. This may be because, in the first stage of the cohort, the risk of infection due to the proximity of a dengue case was assessed. In contrast, in our study, the infection risk was assessed based on its previous serostatus. In addition, it could be explained by the fact that each locality had its own endemic–epidemic transmission dynamics.

We acknowledge the poor clinical characterization of dengue infections occurred in this study as a result of the study design. Another study limitations is the lack of identification of the infecting serotype in assessed individuals; however, although DENV serotype was not detected, the most common circulating serotype was considered the infecting serotype, based on the report by the Sistemas de Vigilancia Epidemiológica [Epidemiological Surveillance Systems]^15^. The study area has been exposed to all DENV serotypes since 1984. Although in the particular period of study there was essentially DENV-1 circulation, with an anecdotic circulation of DENV-2 after 2016^15^. Likewise, a previous work carried out in the same study area in order to determine the serological profile against DENV of the subjects living in these communities, showed that the subjects with positive indirect IgG presented, mainly, antibodies against DENV-1 and DENV-2, and that the positivity of DENV-3 and DENV-4 decreases after 25 years of age, which suggests that these two serotypes circulate with less intensity in the area compared to DENV-1 and DENV-2^13^.

As previously noted, we finished the follow-up of this cohort in November 2016, and Zika virus (ZIKV) transmission was initiated in Mexico early in 2015, but just one zika case was registered in the study area at the end of August 2016^15^. We acknowledge the general idea about the limitation of assessing recent DENV infection by serology in a region where more than one flavivirus circulates. Also, it is well known the limitations on interpretation of IgM/IgG capture ELISA kit by PANBIO to diagnose DENV recent infection, specifically in regions where ZIKV are co circulating. Having that in mind, we must differentiate the specificity of each test, that is: IgM capture ELISA specificity 80–85%, and IgG capture ELISA specificity 40–50%^30,31^. For these reasons, the recent DENV infections were confirmed by the presence or absence of IgM and/or IgG antibodies until May 2015, and after this date (follow-up 6 and 7), the DENV infections were confirmed only by detection of IgM immunoglobulin^32,33^.

Furthermore, in order to evaluate if IgM capture assay was suitable to reach the conclusions of this study, we assumed that, at most, 20% of the samples could have been false positives by ZIKV cross-reactivity^30^. We decided to subtract this proportion to the observed incidence and rerun the Cox proportional hazards model; the rationale was, if using 20% false positives as the stringent scenario, and the tendency is conserved, we can rule out an influence of zika antibodies. It was observed that the protection tendency was conserved although the significance was lost (p > 0.05) (Supplementary Table S3).

Altogether, we are confident on the results because the localities of the study were essentially free of zika based on the epidemiological surveillance, the recent DENV infections were confirmed considering the potential cross-reactivity, and the risk tendency remained in the sensitivity analysis.

In conclusion, this prospective study assayed immune responses to classify participants according to their infection history, building on the first stage of the cohort and identifying previous serostatus as a protective factor for new DENV infection in an endemic Mexican population.

Our research group has been interested in characterizing the dengue endemic nature and which are the drivers that determine it. In particular, in this project, we were interested in observing how immunity against DENV determines the incidence of infection. Although it was not our objective, these results are in line with observations that have been aroused around clinical studies of vaccination with various antigens and the discussion of the best vaccination strategy, for example, recently Hou et al. demonstrated that sequential vaccination generates robust antibody and immune cell response^34^; likewise, Macias et al. have suggested that vaccination with the TAK-003 vaccine followed by DENGVAXIA could improve the performance of the two separate vaccines^35^. What is implicit in both papers is that reinfection by a different DENV antigen determines the protection of the individual, so this work contributes to the discussion by providing evidence in this regard.

## Methods

### Study design and population

The present study is an extension of the prospective cohort study “Peridomestic Infection as a Determinant of Dengue Virus Transmission”, in localities of Axochiapan and Tepalcingo, of the State of Morelos, Mexico. These localities are located at 1030 m above sea level (masl) with 17,508 inhabitants and 1160 masl with 12,053 inhabitants, respectively^36^.

The first stage of the cohort study initiated in 2011 and finished in 2012^14^. Briefly, the study included participants living near a index dengue case (exposed cohort) and participants living in areas where no dengue cases were reported within 100-m radius in the 2 months previous to the sample day (unexposed cohort). Methodological details of the first part of the study can be found in Martinez-Vega et al.^37^. The study population from the first stage of the cohort included 1196 participants of 5-year-old or older from the localities. The results of this stage of the cohort were necessary to define recent infection and to generate groups in the second stage of the study, according to immune status to DENV.

The second stage of the study of this cohort started in August 2014 and ended in December 2016, in order to assess infection history and its protective effect. The inclusion criteria in the second stage of the cohort were the following: participants who belonged to the abovementioned cohort (1196) or who were relatives of participants and signed the informed or explicit consent form for this new cohort stage. Participants who did not plan to live in the study location during the follow-up period were excluded. Home visits were performed every 6 months (5 visits total). During home visits, a questionnaire was administered and a venous blood sample was taken.

Between each follow-up, an active surveillance were carried out just by telephone calls once a month, to ascertain whether febrile events have taken place among the participants (Fig. 1).

Further, it is important to mention that there is no report of flavivirus circulation other than DENV before 2015, and on the other hand, in Mexico, there is no mandatory vaccination against Yellow Fever or Japanese Encephalitis^38^.

### Laboratory assays

The serum samples were stored at − 70 °C. The seroprevalence was assessed in the cohort by dengue IgG-Indirect ELISA (E-DEN 01 G; Pan Bio, Abbott., Freehold, NJ, USA) following the indications of the manufacturer. The ELISA has 91.5% agreement with respect to Foci Reduction Neutralization Test (FRNT); overall it has 98% specificity and 100% sensibility^39^.

The diagnostic DENV recent infection was performed using the IgM and IgG capture ELISA PANBIO test (Cat No. E-DEN02G, and E-DEN01 M). For IgM, the sensitivity and specificity were 87% and 96% respectively, and for IgG the sensitivity was 100% and the specificity was 98%^40^. Because starting 2016, ZIKV transmission was reported in the area, we discard the IgG capture assay to determine recent DENV infection in that year, we still diagnostic recent DENV infection by IgM capture assay for the reason mentioned next.

### DENV infection definition

The sample was considered a recent infection by DENV if the sample was positive for IgM. In case that the sample was negative for IgM-Capture but positive for IgG-Capture, it was considered the ratio of PANBIO units for IgG, obtained during the measurement in the first and second follow-up of the first stage of the study.

The previous decision was taken according to the observations made between participants infected and noninfected to DENV; in the group, with recent infection, the ratio of PANBIO units, was greater than one, and in the uninfected group, the ratio was less than one (Table 4).

**Table 4:** Evaluation of the concordance between diagnosis by laboratory and the algorithm created with respect to the ratio of PANBIO Units of IgG.

Lab test									
Baseline (first follow-up)		Second follow-up		Samples with IgG2/IgG1 > 1	Mediana (units PANBIO)	Range (units PANBIO)		Diagnostic infection by seroconversion	% of agreement^a^
IgM	IgG	IgM	IgG	IgG2/IgG1					
Negative	Negative	Positive	Negative	N.A.^b^	N.A.^b^	N.A.^b^	N.A.^b^	12	N.A.^b^
Negative	Negative	Positive	Positive	5	22.08	9.24	37.22	5	100
Negative	Negative	Negative	Positive	20	7.30	1.04	57.28	20	100

^a^The percentage of agreement was made between the diagnosis by laboratory and the algorithm created with respect to the ratio of PANBIO units.

^b^*N.A*. not apply.

### Definition of recent infection by DENV between 2015 and 2016

According to the Epidemiological Surveillance System of the Ministry of Health of Mexico, the transmission of ZIKV in the state of Morelos began during epidemiological week 32 (August 7–13 of 2016); therefore, we consider that the area of study was essentially free of circulation of ZIKV, until the 6th follow-up of our study (January–July 2016). The case of zika reported in the study area occurred on August 30, 2016, that is, at the beginning of the last follow-up stage.

It is possible that ZIKV transmission started before the case was detected, but we conclude that is not the case for the following reasons: (1) there was no report of another cases in the following 5 months, (2) IgM reactivity on IgG + capture cases remains negative in samples taken before 2016 (Supplementary Fig. S1A); (3) IgM capture remains negative in participants diagnosed by IgG indirect seroconversion before 2016 (Supplementary Fig. S1B).

For follow-ups from August 2015 to November 2016, recent DENV infections were only diagnosed if IgM was positive, in order to exclude possible false positives from cross-reaction of IgG for ZIKV because of the circulation of this virus in the area.

### Compare groups according to immune status previous to DENV infection

To identify immune state factors related to a recent infection by DENV, the participants were classified into four groups:

1. Seronegative group: participants with a negative IgG-indirect test on 2014.
2. Seropositive with at least two previous infections: participants with a positive IgG-indirect test at baseline (2011) and with recent infection detected at the second follow- up.
3. Seropositive with at least one previous infection (between 2012 and 2014): participants with a negative IgG-indirect test at 2012, but a positive IgG-indirect test in 2014, which means that these participants had a primary infection between the first and second stage of the cohort.
4. Participants with a positive IgG-indirect test at baseline (2011) without recent infection at baseline or during the second follow-up (2012) were not considered for this study, due to a lack of information on history of infection before of the 2011.

### Statistical analysis

A descriptive analysis of the sample characteristics was performed using the chi-square test and Fisher’s exact test to compare categorical variables, and the Mann–Whitney test for continuous variables.

The dengue incidence per 1000 person-years was calculated as: 1000 × infections/(sum of the follow-up at-risk period for each individual/365.2).

A Cox proportional hazards model was used for the estimation of hazards related to a recent infection by DENV during the follow-up period. In the Cox proportional hazards model, failures corresponded to all individuals who were diagnosed with a recent infection during the follow-up period between 2014 and 2016, with 2014 (follow-up 3) as time zero. Censored data included all individuals who were not diagnosed with any type of infection during follow-up. The outcome variable was follow-up time (2014–2016) for censored data, and for failures, it was the elapsed time from inclusion in the study in 2014 to the first infection.

**Figure 3:**
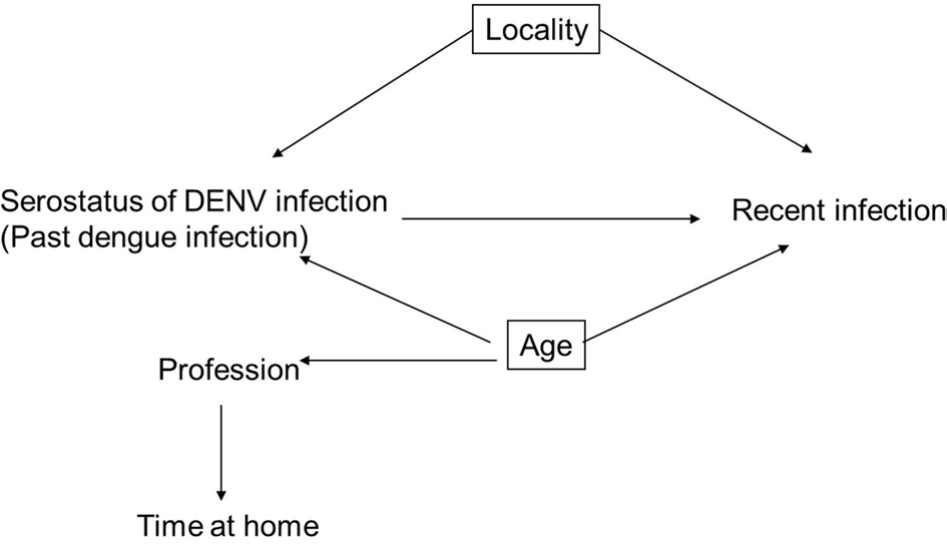
Directed acyclic graph (DAG).

The analysis was controlled for confounders variables included: age and location (Fig. 3).

Besides, standard errors were adjusted by 75 clusters of the sampling cohort, in which each cluster was formed by houses located 50 m around the house of a dengue case reported in 2011. The STATA SE 14 software was used for the statistical analysis.

### Ethical standards

“Peridomestic Infection as Determinant of Dengue Virus Transmission” (CI: 986) and the present study (CI: 494) have the authorization of the Ethics Commission of the National Institute for Public Health. Additionally, the study has the signed informed consent form for all adults, parents or guardians of children and the signed informed approval form for people under 18 years old.

## Supplementary information

Supplementary Information

## Data Availability

The database is unavailable since this project is covert by the Mexican FEDERAL LAW ON PROTECTION OF PERSONAL DATA HELD BY PRIVATE PARTIES (information about the law can be found in: https://privacyassociation.org/media/pdf/knowledge_center/Mexico_Federal_Data_Protection_Act_July2010.pdf). For specific inquiries about the data, interested subjects can contact Ms. Angelica R Angeles-Llerenas, Chair of the Institutional Ethics Board (aangelica@insp.mx).
