# Supplementary material for: Risk of dengue virus infection according to serostatus in individuals from dengue endemic areas of Mexico
Source: Sci Rep. 2020 Nov 4;10:19017. doi: 10.1038/s41598-020-75891-z (PMC7642410; doi:10.1038/s41598-020-75891-z)
Supplement: Supplementary file 1 — Supplementary Information [file 41598_2020_75891_MOESM1_ESM.docx]

RISK OF DENGUE VIRUS INFECTION ACCORDING TO SEROSTATUS IN INDIVIDUALS FROM DENGUE ENDEMIC AREAS OF MEXICO

I.Y. AMAYA-LARIOS^,2^ R.A. MARTÍNEZ-VEGA^3,4^ F.A. DIAZ-QUIJANO^5^ E. SARTI^6^ E. PUENTES-ROSAS^6^, L. CHIHU^1^ AND J. RAMOS-CASTAÑEDA^1,7*^

^1^Instituto Nacional de Salud Pública, Mexico. ^2^Consejo Nacional de Ciencia y Tecnología, México. ^3^Universidad de Santander, Colombia. ^4^Organización Latinoamericana para el Fomento de la Investigación en Salud, Colombia. Faculdade de Saúde Pública, Universidade de São Paulo, Brazil ^6^ , Sanofi Pasteur. ^7^Center for Tropical Diseases, University of Texas-Medical Branch. USA.

*Corresponding Author. Centro de Investigaciones sobre Enfermedades Infecciosas. Instituto Nacional de Salud Publica. Av Universidad 655, Santa Maria Ahuacatitlan. Cuernavaca, Morelos, Mexico 62100. jramos@insp.mx

Supplementary Material

**Supplementary Table S1. DENV positive samples from Morelos, México (2014-2016). José Ramos-Castañeda, unpublished data.**

| Year |  | DENV | |
| --- | --- | --- | --- |
|  |  | 1 | 2 |
| 2014 | % | 93.6 | 6.4 |
|  | n | 29 | 2 |
| 2015 | % | 66.7 | 33.3 |
|  | n | 14 | 7 |
| 2016 | % | 57.7 | 42.3 |
|  | n | 56 | 41 |

% : Percent; n: Number of samples

**Supplementary Table S2: Classification of recent DENV infections according to the results of diagnostic tests by follow-up stage.**

| **Follow-up** |  | | | | |
| --- | --- | --- | --- | --- | --- |
|  | **(IgM - IgG +)** | **(IgM + IgG +)** | **IgG-Indirect** | **(IgM +)** | **TOTAL** |
| February, 2015 to May, 2015 (Follow-up 4) | 6 | 1 | 0 | 0 | 7 |
| August, 2015 to November, 2015. (Follow-up 5) | 0 | 0 | 2 | 6 | 8 |
| February, 2016 to May, 2016  (Follow-up 6) | 0 | 0 | 0 | 6 | 6 |
| August, 2016 to November, 2016.  (Follow-up 7) | 0 | 0 | 5 | 22 | 27 |
| TOTAL | 6 | 1 | 7 | 34 | 48 |

**Supplementary Table S3. Factors related to incident DENV infection in cohort participants from Axochiapan and Tepalcingo, Morelos (2014-2016), considering an 80% sensitivity of the IgM test.**

|  | HR* | Robust Standard Error | p>\|z\| | 95% Confidence Interval | |
| --- | --- | --- | --- | --- | --- |
| Group |  |  |  |  |  |
| Seronegative | 1 |  |  |  |  |
| Seropositive with at least one previous infections | 1.01 | 0.430 | 0.997 | 0.44 | 2.32 |
| Seropositive with at least two previous infections | 0.58 | 0.221 | 0.150 | 0.27 | 1.22 |
| Age | 1.01 | 0.007 | 0.122 | 1.00 | 1.03 |
| Locality |  |  |  |  |  |
| Tepalcingo | 1 |  |  |  |  |
| Axochiapan | 0.51 | 0.177 | 0.054 | 0.26 | 1.01 |

* HR: Hazard Ratio


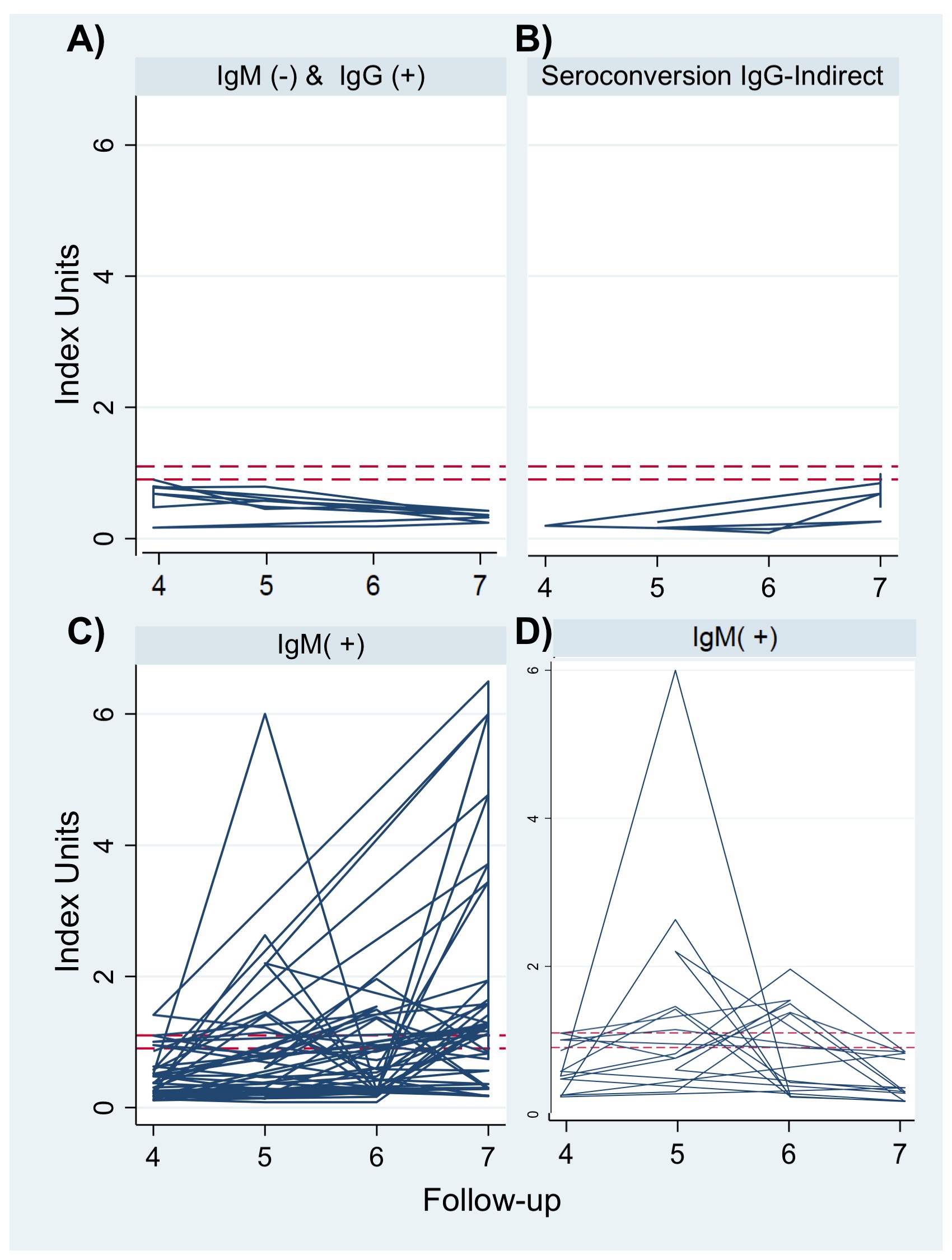


**Supplementary Figure S1: IgM reactivity through time in sera of subjects diagnosed as DENV infected. IgM reactivity among subjects who have an IgG(+) capture reaction (A); IgM reactivity among subjects who seroconvert according to IgG indirect positive reaction (B); IgM reactivity in participants diagnosed as DENV infected (C); (D) reactivity of the subjects that where diagnosed infected in the 4-6 follow up (C) and shows no persistence of IgM reactivity.**
